# Case report: Utilizing diffusion-weighted MRI on a patient with chronic low back pain treated with spinal cord stimulation

**DOI:** 10.3389/fnimg.2023.1137848

**Published:** 2023-05-16

**Authors:** Isaiah Ailes, Mashaal Syed, Caio M. Matias, Laura Krisa, Jingya Miao, Anish Sathe, Islam Fayed, Abdulaziz Alhussein, Peter Natale, Feroze B. Mohamed, Kiran Talekar, Mahdi Alizadeh

**Affiliations:** ^1^Department of Neurosurgery, Thomas Jefferson University, Philadelphia, PA, United States; ^2^Jefferson Integrated Magnetic Resonance Imaging Center (JIMRIC), Department of Radiology, Thomas Jefferson University, Philadelphia, PA, United States; ^3^Sidney Kimmel Medical College, Thomas Jefferson University, Philadelphia, PA, United States; ^4^Department of Occupational Therapy, Thomas Jefferson University, Philadelphia, PA, United States; ^5^College of Osteopathic Medicine of the Pacific, Western University of Health Sciences, Pomona, CA, United States

**Keywords:** diffusion-weighted imaging, spinal cord stimulation, neuromodulation, failed back surgery syndrome, gray matter microstructural organization

## Abstract

Diffusion-weighted magnetic resonance imaging (dwMRI) has increasingly demonstrated greater utility in analyzing neuronal microstructure. In patients with chronic low back pain (cLBP), using dwMRI to observe neuronal microstructure can lead to non-invasive biomarkers which could provide clinicians with an objective quantitative prognostic tool. In this case report, we investigated dwMRI for the development of non-invasive biomarkers by conducting a region-based analysis of a 55-year-old male patient with failed back surgery syndrome (FBSS) treated with spinal cord stimulation (SCS). We hypothesized that dwMRI could safely generate quantitative data reflecting cerebral microstructural alterations driven by neuromodulation. Neuroimaging was performed at 6- and 12- months post-SCS implantation. The quantitative maps generated included diffusion tensor imaging (DTI) parameters; fractional anisotropy (FA), axial diffusivity (AD), radial diffusivity (RD), and mean diffusivity (MD) computed from whole brain tractography. To examine specific areas of the brain, 44 regions of interest (ROIs), collectively representing the pain NeuroMatrix, were extracted and registered to the patient's diffusion space. Average diffusion indices were calculated from the ROIs at both 6- and 12- months. Regions with >10% relative change in at least 3 of the 4 maps were reported. Using this selection criterion, 8 ROIs demonstrated over 10% relative changes. These ROIs were mainly located in the insular gyri. In addition to the quantitative data, a series of questionnaires were administered during the 6- and 12-month visits to assess pain intensity, functional disability, and quality of life. Overall improvements were observed in these components, with the Pain Catastrophizing Scale (PCS) displaying the greatest change. Lastly, we demonstrated the safety of dwMRI for a patient with SCS. In summary, the results from the case report prompt further investigation in applying dwMRI in a larger cohort to better correlate the influence of SCS with brain microstructural alterations, supporting the utility of dwMRI to generate non-invasive biomarkers for prognostication.

## Introduction

The National Health Interview Survey reported that 39% of adults experienced chronic low back pain (cLBP), and 36.5% experienced chronic lower limb pain in the United States in 2019 (Lucas et al., 2019). In some cases, surgical intervention does not alleviate pain and may in fact exacerbate symptoms. These patients can be classified as having failed back surgery syndrome (FBSS), defined as suffering from persistent pain in the lumbar region despite surgery thought likely to bring relief (Thomson, 2013). Even if surgery has failed to alleviate the pain, opioids, NSAIDS and other pharmaceutical treatments may still result in low patient satisfaction (Kumar et al., 2007). Additionally, seeking alternative therapies like physical therapy, acupuncture or transcutaneous nerve stimulation may still result in unsatisfactory pain management. However, after 12–16 weeks of failed conventional medical management, patients can elect to pursue spinal cord stimulation (SCS), which may provide pain relief (Stanton-Hicks, 2006). SCS employs an implantable device that delivers electrical stimulation to nerves in the spinal region, interfering with and reducing neuropathic pain signaling (Sdrulla et al., 2018). These devices have been shown to be effective, with Class I evidence for treating FBSS (Kumar et al., 2007).

The clinical efficacy of SCS has demonstrated a reduction in pain intensity, increased quality of life, and improvements in functional capacity (Kumar et al., 2007). Furthermore, anatomical and functional alterations in the brain have been revealed using various techniques, namely electroencephalography, positron emission tomography, and functional magnetic resonance imaging (fMRI) (Bentley et al., 2016; Goudman et al., 2020). Despite the current limited number of studies, SCS showed effects on functional activity in the insula, medial thalamus, anterior cingulate cortex, and somatosensory cortex (Bentley et al., 2016; De Groote et al., 2019)**;** volumetric changes of gray (GM) and white matter (WM) were observed in the frontotemporal and frontoparietal region, respectively (De Groote et al., 2020a).

Diffusion-weighted magnetic resonance imaging (dwMRI) is another useful, non-invasive technique used to observe GM and WM by assessing neuronal microstructure such as membranous compartments, myelination, or directionality. dwMRI can also generate quantitative data, serving as an objective assessment of GM and WM integrity. Previous studies have utilized dwMRI to investigate patients with cLBP compared to healthy controls (Vachon-Presseau et al., 2016; Mao et al., 2022) and the effects of alternative treatments on brain GM and WM (Kim et al., 2020). To this date, no studies have performed dwMRI to assess the effects of long-term SCS. Therefore, we aim to first demonstrate the safety of 3T dwMRI in an FBSS patient with a permanently implanted SCS system. Second, we hypothesize that brain GM microstructure continues to change with active SCS modulation, which can be detected using dwMRI-based measurements. In this case report, we performed 3T dwMRI on a single FBSS patient at 6- and 12- months after SCS implantation and compared measurements between the two timepoints. Clinical assessments were also collected and compared.

## Case description

The patient is a 55-year-old male whose primary concern was back pain. He reported burning pain throughout the day that localized to his low back and thighs. Additionally, the pain was mildly worsened with movement and disrupted his sleep. In addition to FBSS, the patient's past medical history was significant for fibromyalgia, restless leg syndrome, chronic radiculopathy, obstructive sleep apnea (OSA), anxiety, and depression. Past surgical history included C5-T1 anterior fusion, C3-T1 posterior fusion, and two L4-S1 lumbar fusions.

**Figure 1:**
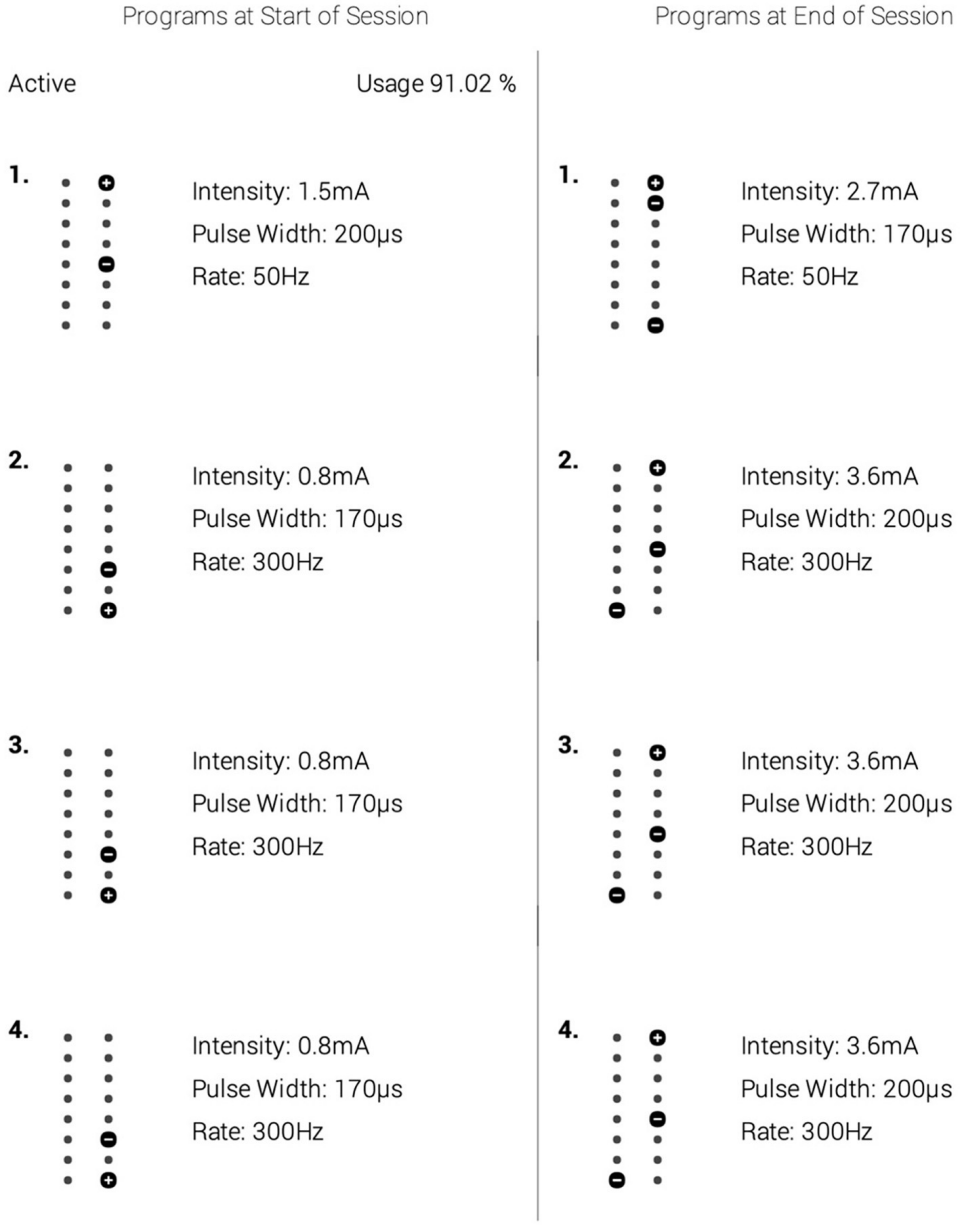
A depiction of the spinal cord stimulation (SCS) parameters that were optimized for the patient's treatment. The left column depicts the settings used for the first 9 months. The right column shows the parameters used in the next 3 months leading to the 12-month follow-up. Within each column are alternative settings. To deliver its analgesic effect, the spinal cord stimulator cycled between settings 1–4 within the respective column. Though the parameters were increased to improve the patient's therapeutic relief, no other intervention was provided at this time.

Conventional medical management for his pain included physical therapy, celecoxib (200 mg twice a day), pregabalin (225 mg twice a day), hydromorphone (2 mg as needed), and Tizanidine (4 mg as needed) but was unsuccessful in alleviating pain. However, these medications were taken over a 20-year period. At this time, the patient had a visual analog scale (VAS) score of 7/10 reflecting their level of current pain, with 0 being no pain and 10 being extreme pain. Given his condition, he elected to pursue an 8-day trial of SCS which was successful since he reported 100% pain relief, no side effects, and had a VAS of 0/10. Afterward, the patient received permanent SCS implantation [*Intellis*™ *with AdaptiveStim*™ *implantable neurostimulator (Medtronic, Inc., Minneapolis, MN, USA)*]. The procedure included the insertion of a Medtronic SCS paddle electrode spanning over T8 and T9 vertebral bodies, in addition to a left flank incision to implant the rechargeable pulse generator. The device was programmed using the Differential Target Multiplexed paradigm (Figure 1). At the 6- and 12-month periods after SCS implantation, dwMRI was performed and clinical measurements were collected.

## Diagnostic assessment

### Quantitative data acquisition

MRI scans with the SCS device switched off were performed at 6- and 12-months post-SCS implantation using a 3T Siemens Prisma MR scanner (Siemens Healthcare, Erlangen, Germany) with a TR/TX head coil. The parameters were: number of directions = 30, b-value =1000 s mm-2 , b0 images = 2, voxel size = 2 × 2 × 2 mm^3^, slice thickness = 2 mm, TR = 11.4 s, TE = 85 ms, number of averages = 1 and acquisition time = 12:23 mins.

**Figure 2:**
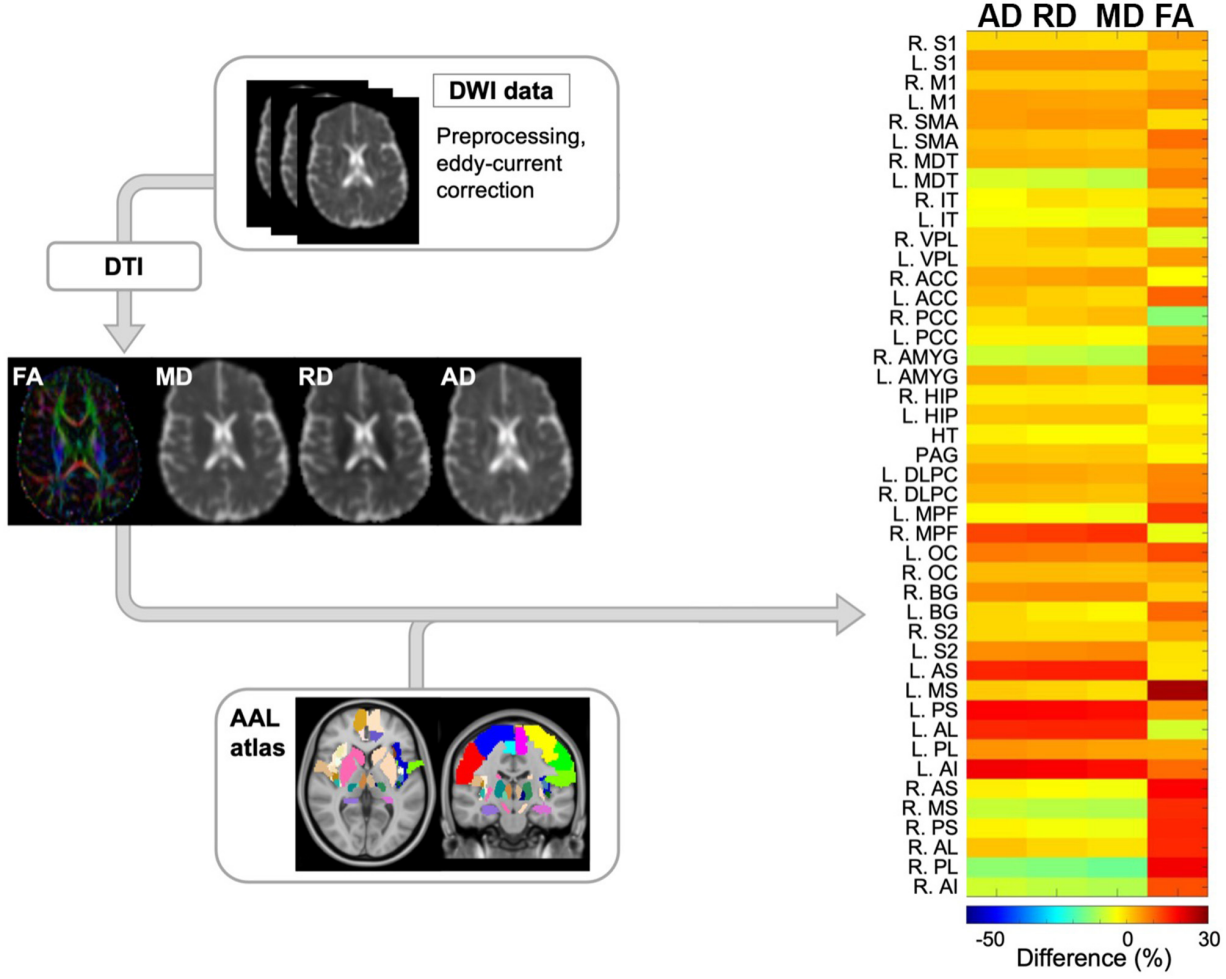
Schematic overview of diffusion-weighted imaging (DWI) processing modules and subsequent computational analysis for anatomical data. Initially, DWI data was preprocessed to correct for eddy current and motion artifacts. Next, tensors were estimated resulting in 4 quantitative maps including fractional anisotropy (FA), mean diffusivity (MD), radial diffusivity (RD), and axial diffusivity (AD). Finally, ROIs from the AAL atlas were registered to the diffusion space and the differences in the aforementioned maps between the 2 timepoints were reported per each ROIs. The representative output of this module is shown as a heatmap (right side) where the main variables of interest across pain-related ROIs are depicted by color. DWI, diffusion-weighted imaging; FA, fractional anisotropy; MD, mean diffusivity; RD, radial diffusivity; AD, axial diffusivity; FOD, fiber orientation distribution; AAL, automated anatomical labeling. NeuroMatrix ROIs are described in the Appendix.

After image collection, dwMRI scans were pre-processed for eddy current distortion, and motion-induced artifacts using FSL FLIRT (FMRIB's Linear Image Registration Tool). Afterwards, eigenvalues from the diffusion tensor matrix were generated from the pre-processed dwMRI volumes using the FSL FDT diffusion toolbox. This led to the generation of diffusion tensor imaging (DTI) maps including fractional anisotropy (FA), axial diffusivity (AD), radial diffusivity (RD), and mean diffusivity (MD) (Figure 2). To examine specific areas of the brain, 44 regions of interest (ROIs) from a customized AAL atlas were extracted and registered to the patient's diffusion space using Medical Image Registration ToolKit (MIRTK) technique. These 44 regions comprise structures integral to the pain NeuroMatrix and are detailed in the Appendix. ROIs were segmented to the patient space leading to the generation of quantitative data at the 6- and 12-month timepoints.

### Clinical assessments

To complement the imaging data, a series of assessments were administered at 6- and 12-months post-SCS implantation. These assessments were performed on the same day of neuroimaging and aimed to determine if SCS may influence pain intensity, functional disability, and quality of life. To assess low back and leg pain intensity, the patient completed the Numeric Pain Rating Scale (NPRS). The NPRS averaged the patient's worst, current, and lowest pain experienced during the past 24 h by using a scale from 0 (no pain) to 10 (extreme pain).

To assess functional disability, the patient completed the Central Sensitization Inventory (CSI). The CSI assessed if the patient was experiencing symptoms due to central sensitization syndromes, with each question scored from 0 (never) to 4 (always). Scores greater than 40 are deemed significant for symptoms due to central sensitization. Another questionnaire utilized was the Oswestry Disability Index (ODI). The ODI assessed the degree of disability due to pain with each question scored from 0, expressing normal autonomy, to a score of 5, outlining complete disability.

To assess quality-of-life, the Pain Catastrophizing Scale (PCS) entailed questions aiming to elucidate thoughts and feelings invoked by pain. Each question was scored from 0 (not at all) to 4 (all the time) depicting to what extent negative thoughts or feelings arose when in pain. The next assessment was the Pittsburgh Sleep Quality Index (PSQI) which served to evaluate the quality of sleep and its impact on daily activities. Specifically, 7 components were scored from 0 to 3: subjective sleep quality, sleep latency, sleep duration, habitual sleep efficiency, sleep disturbances, sleep medications, and daytime dysfunction. Higher scores represent worse sleep quality.

The last assessment for quality of life was the Hospital Anxiety and Depression Scale (HADS). The questions were scored from 0 to a score of 3, interpreted as final scores between 0 and 7 (normal), between 8 and 10 (borderline abnormal), and 11–21 (abnormal).

## Results

### Results of region-based assessment

**Figure 3:**
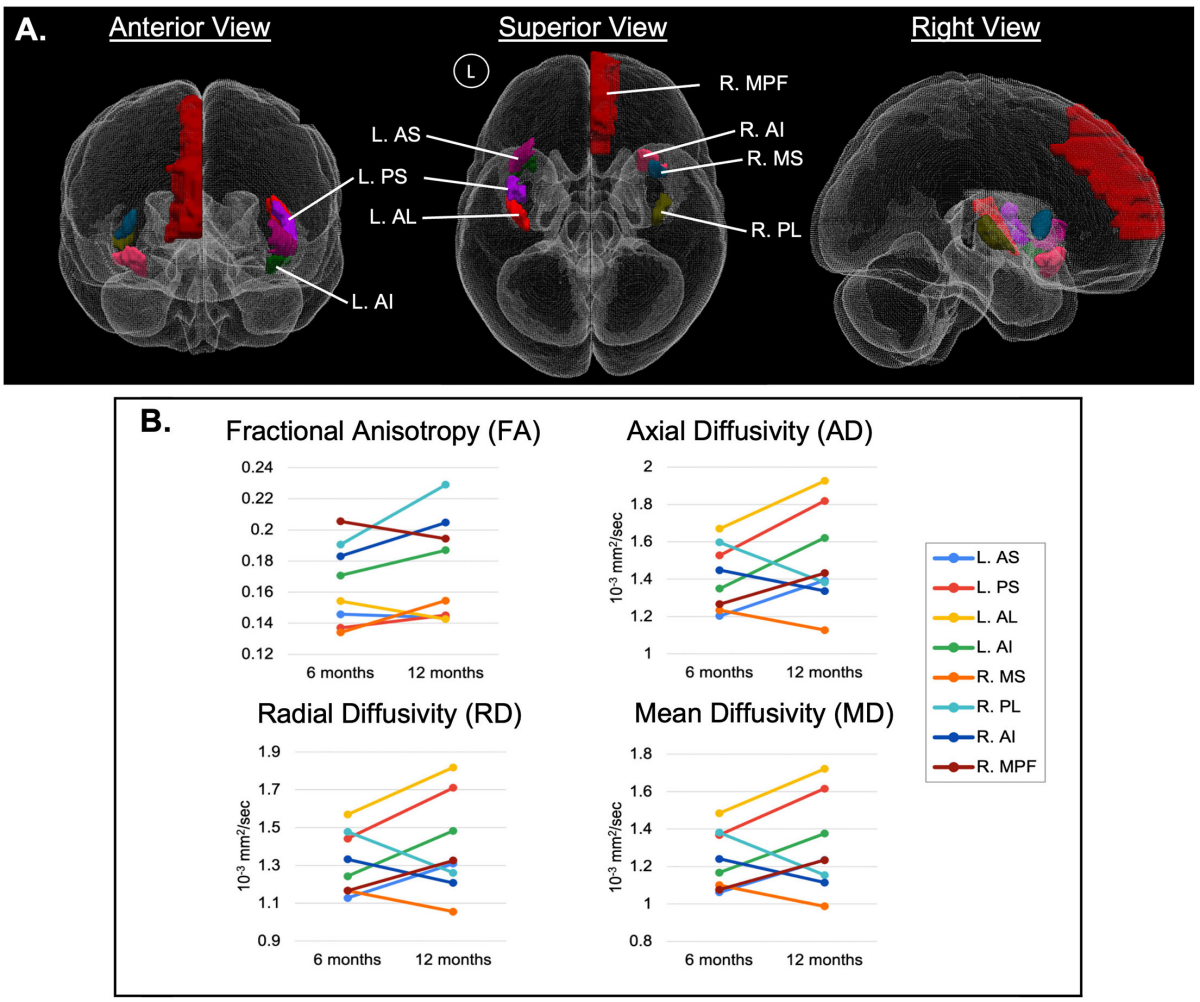
**(A)** 3D representation of significant ROIs, arising from the region-based analysis of diffusion-weighted MRI (dwMRI) in an individual with spinal cord stimulation (SCS) for failed back surgery syndrome (FBSS), predominantly related to the insular gyri. Regions with significant changes were defined as those with >10% relative change in at least 3 of the 4 DTI-derived metrics between the 6- and 12-month periods and are represented by ladder plots in **(B)**. MPF, medial prefrontal cortex; AS, anterior short insular gyrus; MS, middle short insular gyrus; PS, posterior short insular gyrus; AL, anterior long insular gyrus; PL, posterior long insular gyrus; AI, anterior inferior insular gyrus; with cerebral hemispheres denoted as R, right hemisphere and L, left hemisphere.

Regions with significant changes were defined as those with >10% relative change in at least 3 of the 4 DTI-derived metrics between the 6- and 12-month periods. Eight of 44 ROIs had significant changes, including the R.MPF, R.MS, R.PL, R.AI, L.AS, L.PS, L.AL, and L.AI. These ROIs were predominantly related to the insular gyri (Figure 3). In 5 ROIs (R.MPF, L.AS, L.PS, L.AL, and L.AI), the relative change for the DTI indices were positive, whereas 3 ROIs (R.MS, R.PL, and R.AI) had negative changes. This suggests that regions in the left hemisphere had positive DTI changes, while regions in the right hemisphere had mostly negative DTI changes, except for the R.MPF. However, of the 8 regions, only 3 regions in the right hemisphere (R.MS, R.PL, and R.AI) and 1 region in the left hemisphere (L.AI) had a change in FA >10% (Figure 3). Also, a trend in the data showed that AD, RD, and MD had similar relative changes within each region while FA appeared independent from those indices.

### Results of clinical assessments

The clinical assessment data (Table 1) depicted minimal changes in the NPRS. The CSI score decreased by 4 points from 30/100 to 26/100. Moreover, the ODI decreased by 4 points from 10/50 to 6/50. In the quality-of-life category, the PCS score decreased by 17 points from 26/52 to 9/52. The PSQI increased by 1 point from 13 to 14 points. The HADS at 6 months was rated 6 points for anxiety and 7 points for depression. At 12 months, there were 6 points for anxiety and 4 points for depression, netting no change in the anxiety score, but a 3-point improvement in the depression score. Lastly, the patient reported no adverse effects from MRI.

**Table 1:** The clinical assessment results from both the 6-month and 12-month periods post-spinal cord stimulator implantation.

**Questionnaires**	**Scores**		
	**6 months**	**12 months**	Δ **score**
**Low back and leg pain intensity**			
Numeric pain rating scale	3.5	3.6	+0.1
**Functional disability**			
Central sensitization inventory	30/100	26/100	−4
Oswestry disability index	10/50	6/50	−4
**Quality of life**			
Pain catastrophizing scale	26/52	9/52	−17
Pittsburgh sleep quality index	13	14	+1
**Hospital anxiety and depression scale**			
Anxiety	6	7	+1
Depression	6	4	−3

Scoring systems for each of the 6 assessments are as follows: The Numeric Pain Rating Scale averages the patient's worst, current and lowest pain experienced during the past 24 hours on a scale from 0 (no pain) to 10 (extreme pain). The Central Sensitization Inventory scores each of 25 questions from 0 (never) to 4 (always), where a summation of scores >40 is deemed significant for symptoms due to central sensitization. The Oswestry Disability Index scores each of 60 questions from 0 (expressing normal autonomy) to 5 (outlining complete disability). The Pain Catastrophizing Scale scores 13 items from 0 (not at all) to 4 (all the time), depicting to what extent negative thoughts or feelings arose when in pain. The Pittsburgh Sleep Quality Index encompasses 7 components scored from 0 to 3: subjective sleep quality, sleep latency, sleep duration, habitual sleep efficiency, sleep disturbances, sleep medications, and daytime dysfunction, where higher scores represent worse sleep quality. The Hospital Anxiety and Depression Scale is a 14-item measure scored from 0 to 3, interpreted as final scores between 0–7 (normal), 8–10 (borderline abnormal), and 11–21 (abnormal).

## Discussion

### Region-based assessment

The intricacies of GM and WM microstructure have major implications in cLBP, particularly in relation to neuropathology (De Groote et al., 2020a; Robertson et al., 2022). Between dysfunctional glial activation leading to increased neuronal death and reduced neuronal regeneration by virtue of aberrant inflammatory processes (Rao et al., 2012; Loggia et al., 2015; Torrado-Carvajal et al., 2021), the integrity of GM and WM endures considerable decline (De Groote et al., 2020a; Robertson et al., 2022). With high sensitivity to neuronal architecture, DTI indices have been evidenced to depict microstructural GM and WM alterations (Frye et al., 2011; Rulseh et al., 2013). In fact, Mansour et al. (2013) compared DTI metrics in patients who recovered from subacute back pain to those who progressed toward chronicity and found evidence of weakened microstructure in chronic pain patients across cortical and subcortical structures (Mansour et al., 2013).

At the microstructural level, FA quantifies the directionality of diffusivity; with increasing FA values associated with increased myelination, heightened axonal packing density, decreased axonal diameter or decreased membrane permeability (Solowij et al., 2017; Wu et al., 2017). In considering that 5 of the significant ROIs demonstrated an increase in FA between the 6-month and 12-month time points (R.AI, L.AI, R.PL, L.PS, R.MS) in our study, a potential increase in myelination throughout these insular gyri may have been driven by SCS therapy. Adjacent efforts have described the contribution of various stimulation techniques toward remyelination following spinal cord injury in animal models (Solowij et al., 2017; Li et al., 2020), which may be suggestive of the impact of myelin plasticity toward promoting functional recovery and as a protective factor against pain (Solowij et al., 2017).

Likewise, SCS may be promoting axonal regeneration in addition to remyelination, as inferred by the observed decreases in MD, RD, and AD in insular ROIs R.AI, R.PL, and R.MS. By definition, an increase in MD has been indicative of reduced WM integrity due to either axonal or myelin degradation (Solowij et al., 2017), and similarly increased RD may correlate with demyelination possibly of pathological origin, an overall loss of axons, or reduced axonal packing density (Solowij et al., 2017). In contrast, decreased AD has typically been viewed as a measure of axonal degeneration, reduced axonal caliber, or less coherent orientation of axons (Winklewski et al., 2018) yet in the study by Xie et al. (2010) AD was not representative of axonal atrophy (Xie et al., 2010). As such, the decreased MD, RD, and AD values encountered in this analysis may suggest improvement in axonal and myelin integrity in these particular ROIs (Kumar et al., 2013).

In their analysis of 22 FBSS patients, De Groote et al. (2020b) reported volumetric changes in GM and WM following 3-months of SCS therapy, significantly correlating to improved pain relief (De Groote et al., 2020b). The group also detected no differences in 11 patients with FBSS between GM volumes before implantation and after 1-month of SCS, nor between the volumes at 1 and 3 months of SCS (De Groote et al., 2020a,b). Based upon their findings, it is plausible that the microstructural alterations observed in this study had begun to manifest around the 3-month mark of SCS therapy as well; and as evidenced by our imaging at 6- and 12- months post-implantation, there continue to be physiological changes in the neural network due to SCS. As mentioned in Figure 1, the SCS parameters were optimized for the patient's treatment protocol 9-months following implantation. Although frequency (“rate,” Hz) was unchanged, there was an increase in intensity and pulse width. While continued neurophysiological modifications are anticipated due to SCS therapy in general (Peña Pino et al., 2020), there is a possibility that the increase in these metrics enhanced the rate of change of said microstructural alterations, as reflected via the DTI indices explored in Figure 3.

### Involvement of insula in relation to pain

The observed differences in DTI indices ranging across the various, distinct insular gyri may in turn be suggestive of complex functional and structural interrelationships within the insular cortex (Uddin et al., 2014); potentially warranting independent analyses to investigate the role each plays in pain perception. The anterior insular gyrus, for example, is activated in interoception, subjective awareness, pain perception, and salient stimuli discrimination (Chang et al., 2013; Uddin et al., 2014). In a publication describing the role of the anterior insula, Craig (2009) highlighted the anterior insula's role in interoception and how insular activation strongly correlated with pain perception (Craig, 2009). Additionally, the posterior insular gyrus is responsible for sensorimotor tasks, and language processing (Stancák et al., 2008) and has been shown to play a role in pain sensation (Ostrowsky et al., 2002); fMRI studies have shown greater activation in the posterior insula in response to painful stimuli (Stancák et al., 2008).

In summary, the ROIs mentioned have clear roles in pain modulation or perception and are potentially susceptible to SCS. While the mechanism of action driving these changes is unknown, the literature has shown that chronic pain can alter neurochemistry, glial cell recruitment, and overall microstructure (Ong et al., 2019; De Ridder et al., 2021), and that SCS may be reversing abnormal physiology.

### Clinical assessments

To assess pain intensity, the NPRS had an average score of 3.5 over 6 months, showing a minimal change from 6- to 12 months post-implantation. Although the patient reported mild pain intensity, he returned to normal daily activities with SCS.

Regarding functional disability, the patient's CSI scores slightly improved. The patient does not suffer from central sensitization as his score was below the clinical threshold of 40. However, the patient scored worse in questions such as having low energy and feeling sad or depressed, which may be attributable to the patient's OSA.

ODI scores were 20% at 6 months and 12% at 12 months, both of which signified minimal disability. The 4-point reduction in ODI was insignificant as the minimal clinically important difference is a 10-point improvement from baseline 73. However, it demonstrated a 4-point improvement in his sex life and a 1-point improvement in his sleeping habits due to pain reduction.

For the quality-of-life category, the PCS decreased by 17 points from 26/52 at 6-months to 9/52 at 12-months. Further investigation showed the patient was experiencing a flare in his cervical radiculopathy before completing the questionnaire which cannot be treated with lumbar SCS. Similarly, the PSQI scores at 6- and 12-months were indicative of poor sleep quality. However, the reduced quality of sleep was not due to pain but other factors such as OSA. Lastly, the HADS showed a 3-point improvement in depression due to increased enjoyment in life and a reduced sensation of tension.

### Limitations

Our results were limited by the lack of a preoperative assessment which would have served as a baseline for the 6- and 12-month periods. This would have better highlighted changes in microstructural alterations due to neuromodulation, while also improving the clinical assessments. Additionally, the patient had other comorbidities like OSA, depression, and anxiety which can impact the clinical assessments. Nevertheless, dwMRI quantitative analysis is underexplored and deserves further investigation. We intend to continue exploring dwMRI in cLBP patients treated with SCS and are striving toward increasing participant recruitment; both those persons with cLBP following FBSS, as well as healthy controls.

## Conclusion

This case report explored the utility of dwMRI in an FBSS patient treated with SCS for his cLBP. Recruiting this patient provided newfound data showing the ability to safely develop non-invasive biomarkers which highlight alterations in neuronal microstructure. Specifically, notable changes in diffusion properties occurred within the insular gyri and the prefrontal cortex. Though interpretation of the microstructural alterations requires further characterization, promoting the use of dwMRI for quantitative analysis is vital to understanding the potential impact dwMRI may have for developing non-invasive biomarkers in various patient populations.

## Data availability statement

The original contributions presented in the study are included in the article/supplementary material, further inquiries can be directed to the corresponding author.

## Ethics statement

The studies involving human participants were reviewed and approved by the Theodore Taraschi Jefferson Institutional Review Boards. The patients/participants provided their written informed consent to participate in this study. Written informed consent was obtained from the participant/patient(s) for the publication of this case report.

## Author contributions

IA: drafting the manuscript and editing the manuscript. JM: figures and editing the manuscript. AS, LK, FM, AA, IF, CM, PN, MS, and KT: editing the manuscript. MA: principal investigator and editing the manuscript. All authors contributed to the article and approved the submitted version.

## Appendix

**Table A1:**

**Abbreviation**	**Region of Interest (ROI)**
S1	Primary Somatosensory Cortex
S2	Secondary Somatosensory Cortex
M1	Primary Motor Cortex
ACC	Anterior Cingulate Cortex
PCC	Posterior Cingulate Cortex
SMA	Supplementary Motor Area
BG	Basal Ganglia
MPF	Medial Prefrontal Cortex
DLPC	Dorsolateral Prefrontal Cortex
HT	Hypothalamus
PAG	Periaqueductal Gray
AMYG	Amygdala
MDT	Mediodorsal Thalamus
IT	Intralaminar Thalamus
VPL	Ventral Posterolateral Nucleus
HP	Hippocampus
OC	Orbitofrontal Cortex
AS	Anterior Short Insular Gyrus
MS	Middle Short Insular Gyrus
PS	Posterior Short Insular Gyrus
AL	Anterior Long Insular Gyrus
PL	Posterior Long Insular Gyrus
AI	Anterior Inferior Insular Gyrus
R	Right Hemisphere
L	Left Hemisphere
